# Substrate Specificity Provides Insights into the Sugar Donor Recognition Mechanism of *O*-GlcNAc Transferase (OGT)

**DOI:** 10.1371/journal.pone.0063452

**Published:** 2013-05-21

**Authors:** Xiaofeng Ma, Pi Liu, Hui Yan, Hong Sun, Xiaoyan Liu, Feng Zhou, Lei Li, Yi Chen, Musleh M. Muthana, Xi Chen, Peng G. Wang, Lianwen Zhang

**Affiliations:** 1 College of Pharmacy, State Key Laboratory of Medicinal Chemical Biology and Tianjin Key Laboratory of Molecular Drug Research, Nankai University, Tianjin, China; 2 Department of Chemistry, University of California Davis, Davis, California, United States of America; Oak Ridge National Laboratory, United States of America

## Abstract

*O*-Linked β-*N*-acetylglucosaminyl transferase (OGT) plays an important role in the glycosylation of proteins, which is involved in various cellular events. In human, three isoforms of OGT (short OGT [sOGT]; mitochondrial OGT [mOGT]; and nucleocytoplasmic OGT [ncOGT]) share the same catalytic domain, implying that they might adopt a similar catalytic mechanism, including sugar donor recognition. In this work, the sugar-nucleotide tolerance of sOGT was investigated. Among a series of uridine 5′-diphosphate-*N*-acetylglucosamine (UDP-GlcNAc) analogs tested using the casein kinase II (CKII) peptide as the sugar acceptor, four compounds could be used by sOGT, including UDP-6-deoxy-GlcNAc, UDP-GlcNPr, UDP-6-deoxy-GalNAc and UDP-4-deoxy-GlcNAc. Determined values of Km showed that the substitution of the N-acyl group, deoxy modification of C6/C4-OH or epimerization of C4-OH of the GlcNAc in UDP-GlcNAc decreased its affinity to sOGT. A molecular docking study combined with site-directed mutagenesis indicated that the backbone carbonyl oxygen of Leu653 and the hydroxyl group of Thr560 in sOGT contributed to the recognition of the sugar moiety via hydrogen bonds. The close vicinity between Met501 and the N-acyl group of GlcNPr, as well as the hydrophobic environment near Met501, were responsible for the selective binding of UDP-GlcNPr. These findings illustrate the interaction of OGT and sugar nucleotide donor, providing insights into the OGT catalytic mechanism.

## Introduction

*O*-GlcNAc modification, namely *O*-GlcNAcylation, is an essential post-translational modification with a single β-*N*-acetylglucosamine linked to Ser or Thr residues of various nucleocytoplasmic proteins [1], [2]. It contributes to various cellular cascades, including signal transduction [3]–[5], gene expression [6], [7] and protein trafficking [8]. Dysregulation in O-GlcNAcylation is assumed to be tightly linked to chronic diseases, such as cancers [9]–[11], diabetes [12] and neurodegenerative diseases [13], [14].

O-GlcNAcylation is mediated by the unique pair of enzymes *O*-GlcNAc transferase (OGT) and *O*-GlcNAcase (OGA). OGT catalyzes the transfer of GlcNAc from UDP-GlcNAc to the Ser/Thr residues in a protein or peptide, while OGA is responsible for sugar removal. The human *ogt* gene is located on the X chromosome at position Xq13.1, and three variants (ncOGT, mOGT and sOGT) are produced by alternative splicing during gene expression [15]. Sequence alignment indicates that all the three OGT isoforms comprise mainly two functional regions: an N-terminal tetratricopeptide region (TPR) and a C-terminal multidomain catalytic region. The TPRs, consisting of a varied number of TPR units among different isoforms, are proposed to regulate protein-protein interactions and are associated with substrate specificity of OGT [16]–[19]. The C-terminal region, where the active site lies, is composed of two conservative domains: CDI and CDII [20], [21].

Structural information helps us get deep into the catalytic mechanism of OGT. The first line of structural information arose from a comparative study of sequence-similar proteins: phosphatase and N-GlcNAc transferase, giving a structural model for the TPR domain and catalytic domain, respectively. It is indicated that the C-terminal region of human OGT (hOGT) consists of two Rossmann-like domains and a conserved motif in the second Rossmann domain points to the UDP-GlcNAc donor binding site [22]. In 2004, Jinek et al. reported the crystal structure of the N-terminal TPR domain of hOGT. This indicated that the TPR domain plays an important role in OGT dimerization, as well as its interaction with nup62 and other substrate proteins [23]. Later, a bacterial OGT from *Xanthomonas campestris* (*Xc*OGT) was co-crystallized with the sugar donor –UDP-GlcNAc [24], [25]. The high sequence similarity between *Xc*OGT and hOGT (up to 36%) allows its application in modeling and mechanism studies for hOGT. The structure in combination with sequence alignment and site-directed mutagenesis illustrates that OGT has a conserved UDP-GlcNAc binding pocket. In hOGT, Lys842/Gln839 was involved in interactions with phosphates, while Asp925/Lys898 might interact with the nucleoside of the sugar donor. Thr202 in *Xc*OGT (corresponding to Thr560 in hOGT) was shown to form a hydrogen bond (H-bond) with the C4-OH of UDP-GlcNAc. In 2011, a truncated hOGT isoform containing the N-terminal 4.5 TPRs and the full length C-terminal domain was co-crystallized with UDP and the CKII peptide [21]. Once again, Lys842/His498/His558 was shown to play an important role in hOGT activity. In addition, an independent mutagenesis analysis on CDI and CDII domains also indicated certain amino acids in this region play crucial roles in OGT activity [20]. Recently, mechanism studies for OGT have been conducted via a crystallographic snapshot method by two research groups [26], [27]. It was found that OGT uses a dissociative S_N_2 mechanism involving electrophilic migration of the anomeric center, which is consistent with previous studies on other glycosyltransferases [28].

In the present work, we describe another way to understand the interaction between OGT and a sugar donor. A strategy combining a substrate screen with molecular docking was used to study the roles of sugar moiety in the substrate recognition of OGT. A series of UDP-GlcNAc analogs was applied to profile donor substrate specificity of sOGT, four of which could be used as donor substrates. Subsequent molecular docking and site-directed mutagenesis analysis indicated that the hydrogen bonds between some residues (Leu653 and Thr560) in the sOGT active site pocket and the hydroxyl groups at C4, C6 of UDP-GlcNAc play crucial roles in sOGT recognition of UDP-GlcNAc. The steric hindrance between the N-acyl group and Met501 and hydrophobic environment near Met501 may also participate in selective binding of UDP-GlcNAc analogs.

## Results and Discussion

### Profiling Sugar Donors of sOGT

Since the catalytic domain is identical for the three isoforms of OGT, they should adopt a same or similar catalytic mechanism, including sugar donor recognition. Considering the facility of the prokaryotic expression system, codon optimized sOGT was expressed in *E. coli*. It was found that codon optimization could markedly improve sOGT expression in comparison with the original gene sequence. After one-step purification, the purity of sOGT reached up to 95% (data not shown). The purified sOGT was concentrated to 1.72 mg/mL.

**Figure 1:**
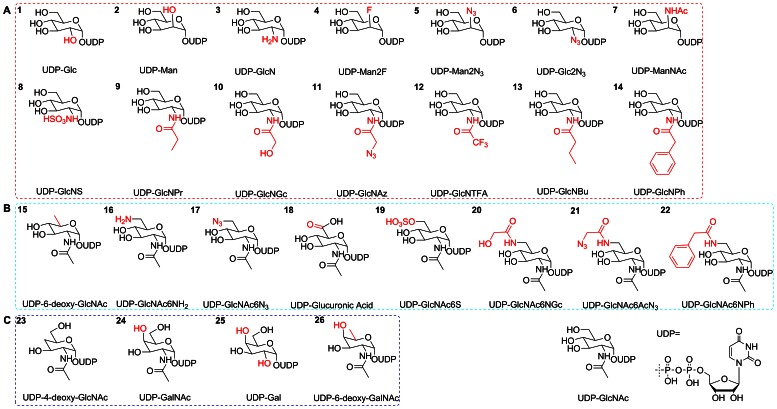
Structures of UDP-GlcNAc analogs used in this work. 14 compounds in the first panel shared the same structure with UDP-GlcNAc except the part at C2 (A). 8 compounds in the second panel were substituted at C6 (B). The rest are UDP-4-deoxy-GlcNAc and UDP-GalNAc derivatives (C). The red part indicates the difference between analog and UDP-GlcNAc.

**Figure 2:**
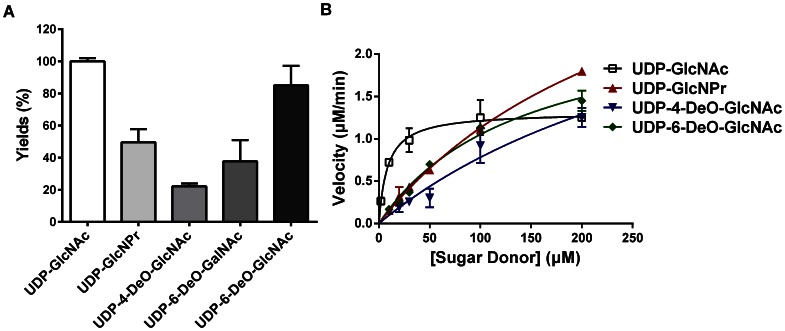
The substrate slectivity of sOGT. (A) The conversion ratio of sOGT with active sugar donors. All reactions were performed under the same conditions. After quenching and removal of proteins, the yields were analyzed with HPLC based on the integrated areas of the products and acceptor substrate. This histogram shows the relative activities of the mutants compared to the wild-type (WT) protein; (B) The Michaelis-Menten curve of sOGT with active sugar donors. Assays were performed using 600 µM CKII peptide and varying concentrations of sugar donors. Reactions were run at 37°C for 30 min with 76 µg of sOGT for the Km measurements. Data were analyzed by Graphpad Prism 5.

A library of 26 UDP-GlcNAc analogs (Figure 1) was applied to evaluate their availability as donor substrates of sOGT. Among these, 14 compounds were substituted at C2, 8 compounds are substituted at C6, and the rest were derived from UDP-GalNAc. Compared with the positive control (UDP-GlcNAc) and negative control (without sugar donor), 4 compounds, including UDP-GlcNPr, UDP-4-deoxy-GlcNAc, UDP-6-deoxy-GalNAc and UDP-6-deoxy-GlcNAc, could be used as active sugar donors for sOGT (Table 1). The different yields for these analogs suggested that they might have different affinity to sOGT (Figure 2A). Herein, in the 14 analogs substituted at the C2 N-acyl group, only UDP-GlcNPr was active. Other compounds, either with a polar substitution or with a bulkier substitution, did not work, indicating that polarity or steric hindrance might affect their recognition by sOGT. Only UDP-6-deoxy-GlcNAc was active in the 8 analogs substituted at C6, and other substitutions with a bulkier group did not work, indicating that an enlargement change at C6 was not bearable. The configuration change of C4-OH to an equatorial bond (UDP-GalNAc) is not active, but an additional C6-deoxy modification (UDP-6-deoxy-GalNAc) made it active again. In addition, the deoxy-analog at C4 (UDP-4-deoxy-GlcNAc) also worked. The results from substrate screening suggested that C4-OH might participate in sOGT recognition of sugar donors and double changes at C4/C6-OH might have changed the spatial conformation of the parent compound and made them flexible to fit into the active site pocket of sOGT.

**Table 1:** List of UDP-GlcNAc analogs in this study and the glycosylation yields.

Entry	Donor Substrate	Yield (%)	Entry	Donor Substrate	Yield (%)
**1**	UDP-Glc	ND^a^	**14**	UDP-GlcNPh	<1
**2**	UDP-Man	ND^a^	**15**	UDP-6-deoxy-GlcNAc	85.08
**3**	UDP-GlcN	ND^a^	**16**	UDP-GlcNAc6NH_2_	ND^a^
**4**	UDP-Man2F	ND^a^	**17**	UDP-GlcNAc6N_3_	<1
**5**	UDP-Man2N_3_	ND^a^	**18**	UDP-Glucuronic Acid	ND^a^
**6**	UDP-Glc2N_3_	ND^a^	**19**	UDP-GlcNAc6S	ND^a^
**7**	UDP-ManNAc	ND^a^	**20**	UDP-GlcNAc6NGc	ND^a^
**8**	UDP-GlcNS	ND^a^	**21**	UDP-GlcNAc6AcN_3_	ND^a^
**9**	UDP-GlcNPr	49.57	**22**	UDP-GlcNAc6NPh	ND^a^
**10**	UDP-GlcNGc	<1	**23**	UDP-4-deoxy-GlcNAc	22.21
**11**	UDP-GlcNAz	<1	**24**	UDP-GalNAc	<1
**12**	UDP-GlcNTFA	<1	**25**	UDP-Gal	ND^a^
**13**	UDP-GlcNBu	ND^a^	**26**	UDP-6-deoxy-GalNAc	37.72

ND^a^: not detected.

Based on the results of substrate screening, we propose that it is stringent for the hydroxyl groups at C4 and C6 during sOGT-sugar donor recognition. In contrast, the C2 N-acyl group can bear certain substitutions, such as in UDP-GlcNPr, though bulkier or hydrophilic substitution could not be accepted. Previous works indicated that UDP-GlcNAz, UDP-GlcNAc6N_3_ and UDP-GalNAc could be used as sugar donors by hOGT [26], [29]–[31]. Moreover, UDP-GlcNAz could be used in the glycosylation of nup62 and a peptide from human α-A crystallin *in vitro* or metabolized onto O-GlcNAcylated proteins *in vivo*, indicating these sugar donors should be acceptable by OGT [32], [33]. The different results in our experiment might be due to the selection of a different acceptor substrate [34]. To test this, these three sugar donors were tested using an octapeptide (YAVVPVSK, derived from protein EMSY, UniProt Q7Z589) as acceptor [35], [36]. It was found that UDP-GlcNAz is active with less product yield than that of UDP-GlcNAc. However, GlcNAc6N_3_ and UDP-GalNAc were still nonreactive (data not shown), indicating sugar donor recognition of OGT is affected by acceptor substrates.

### Measure of the Affinity of Sugar Donors

Km is an inverse measure of the substrate’s affinity for an enzyme –a small Km indicates high affinity and vice versa. Compared with product yields, Km is more suitable to reflect the donor substrate affinity to sOGT. Therefore, we characterized kinetic parameters of active sugar donors, which are summarized in Table 2. Three active sugar donors with higher yields were applied in an apparent kinetic parameters assay, using UDP-GlcNAc as a positive control. Reactions were performed with a fixed concentration of CKII peptide and varying concentrations of sugar donors. The product yield corresponding to each sugar donor was assayed by HPLC, and the data were analyzed with Graphpad Prism 5 by fitting a nonlinear regression analysis for enzyme kinetics (Figure 2B and Table 2). The kinetic constants of sOGT with UDP-GlcNAc is in agreement with the value reported previously [21]. For four active sugar donors, the Km change was consistent with the aspect of yield (*Km*_[UDP-GlcNAc]_ <<*Km*_[UDP-6-deoxy-GlcNAc]_ <*Km*_[UDP-GlcNPr]_ <*Km*_[UDP-4-deoxy-GlcNAc]_). The results further demonstrated that the substitutions at the C2 N-acyl group and C4/C6-OH could affect the affinity of the sugar donor to sOGT, and the change at C4-OH might have a more significant influence on the affinity.

**Table 2:** Apparent kinetic parameters of active sugar donors.

Kinetic Constants	UDP-GlcNAc	UDP-6-DeO-GlcNAc	UDP-GlcNPr	UDP-4-DeO-GalNAc
***Km*** ** (µM)**	8.5±2.36	141.8±28.14	282.1±47.02	369.5±71.58
***Vmax*** ** (µM •min-1)**	1.3±0.078	2.5±0.28	4.3±0.48	3.7±0.59
***Vmax/Km*** ** (min-1)**	0.16±0.034	0.018±0.0016	0.015±0.00084	0.0099±0.00034

### Modeling the Interactions between the Sugar Donor and sOGT

**Figure 3:**
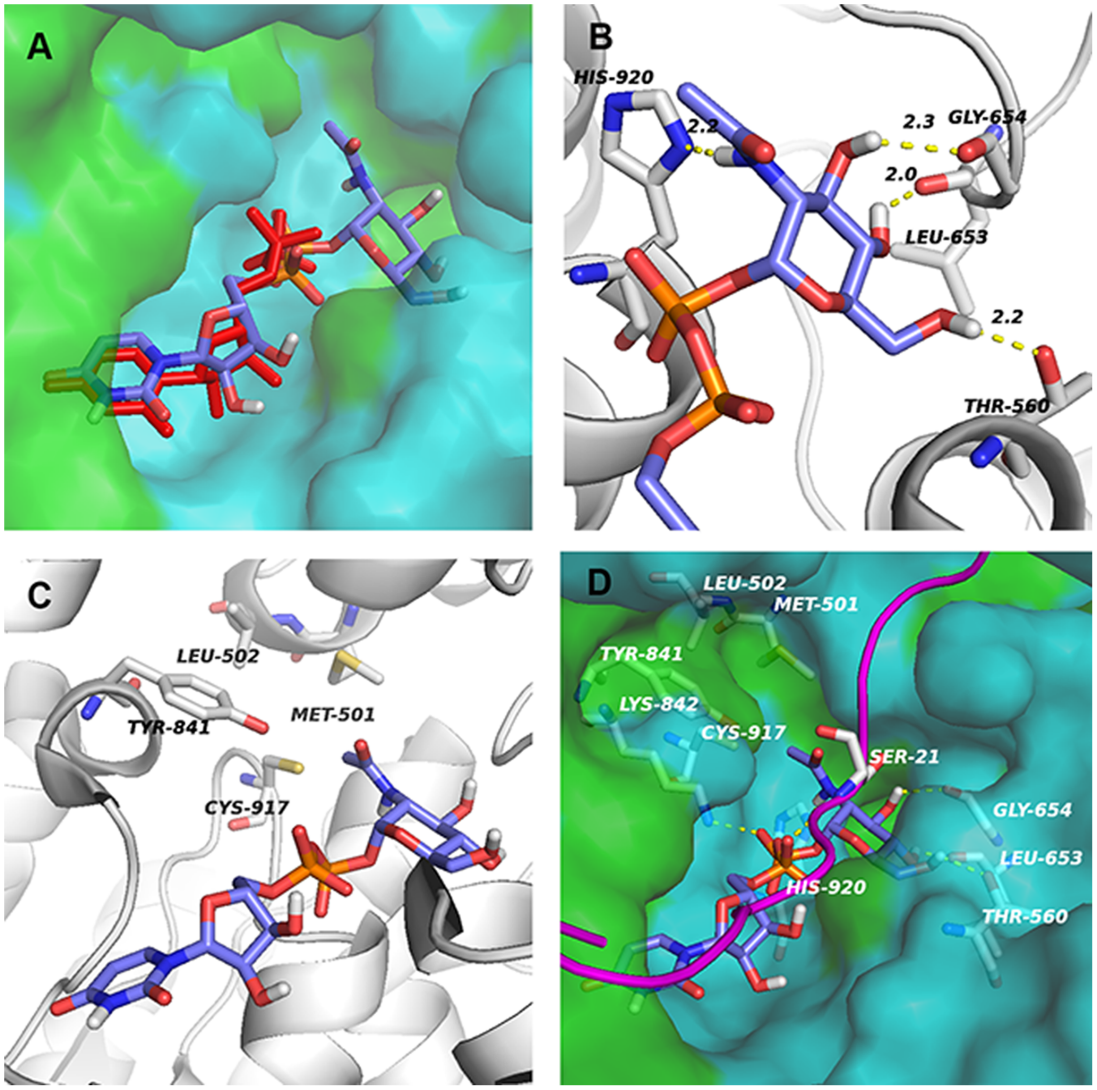
Molecular docking. (A) Co-crystal ligand UDP can be well re-docked into its own binding pocket with RMSD = 0.353 Å. After the re-docking, UDP-GlcNAc was docked into the binding pocket with the co-crystal peptides present, performing the same conformation as UDP. Red stick: co-crystal UDP in 3PE4. Green surface: surface of hydrophobic residues. Blue surface: surface of hydrophilic residues and GLY. (B) There were four possible hydrogen-bond between GlcNAc and transferase; i.e. C6-OH∼Thr560, C4-OH∼Leu653, C3-OH∼Gly654 and the C2 N-acyl group∼His920. (C) The C2-acetamido points to a hydrophobic cave constituted by Met501, Leu502 and Tyr841. (D) There were six possible hydrogen-bonds between UDP-GlcNAc and O-GlcNAc transferase with a peptide substrate. Two of them were from UDP. One was between phosphate and Lys842. The other was between phosphate and Ser21 of the peptide substrate. The yellow dashed lines show the potential hydrogen-bonds. Green surface: surface of hydrophobic residues. Blue surface: surface of hydrophilic residues and GLY. Purple line: CKII peptide. The PyMOL molecular graphics system (Version 1.3r1 Schrödinger, LLC, USA) was used to conduct polarity analysis following manufacturer’s instruction [43].

Obviously, the hydroxyl group changed at C4 and C6, and the hydrophilic or bulkier substitution of the C2 N-acyl group decreased the affinity between the sugar donor and sOGT to some extent. We assume that the hydroxyl group at C4 or C6 may contribute to the binding of OGT and UDP-GlcNAc via a specific H-bond, while the space around the N-acyl group may be insufficient to seat a bulkier group or may not be suitable for the access of a hydrophilic group. To confirm this hypothesis, a molecular docking study was performed using the resolved hOGT structure (PDB ID: 3PE4). Based on the docking parameters obtained from the redocking of UDP (uridine-5′-diphosphate) with the hOGT structure [21], a comparative study of the docking state of UDP and UDP-GlcNAc was processed, showing that UDP-GlcNAc could adopt almost the same conformation as that observed in the reported hOGT-UDP-peptide complex (the RMSD value between the UDP part of UDP-GlcNAc and co-crystal UDP was 1.54 Å; see Figure 3A). In this docking position, close contact was found between C4-OH and the carbonyl oxygen of Leu653, or between C6-OH and the hydroxyl group of Thr560, which renders the formation of H-bond interactions. The hydrogen bond between C4-OH and Leu653 was different from that in *Xc*OGT [24], which might be due to the marked difference of the amino acid sequence between these two OGTs. In addition, two other H-bonds might have formed between C3-OH and Gly654, and between C2-acetamido and His920 (Figure 3B). There was not sufficient space to accommodate a bulkier group substitution in these positions. The N-acyl group of UDP-GlcNAc extended to the Met501 residue in the active site pocket, and limited space was between them (Figure 3C). Polarity analysis of the active site pocket indicated that the space near Met501 was mainly constituted of hydrophobic residues, indicating a hydrophobic substitution of the N-acyl group might be bearable (Figure 3D). The results of molecular docking might provide a reasonable interpretation for either of the changes at C4/C6-OH or at the C2 N-acyl group showing decreased affinity to sOGT.

### Mutagenesis Analysis of Interactions between the Sugar Donor and sOGT

**Figure 4:**
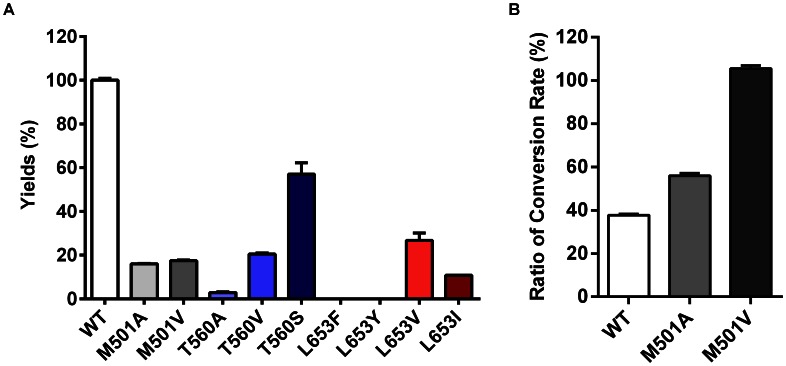
Mutational analysis of key amino acids in sOGT. HPLC analysis of the glycosylation reactions of variant sOGT mutants against UDP-GlcNAc and UDP-GlcNPr. (A) Yields of wild type sOGT and M501A, M501V, T560A, T560V,T560S, L282F, L282Y, L282V and L282I mutants against UDP-GlcNAc. All mutants sustained a great loss in enzyme activity; (B) wild type sOGT and M501A, M501V against UDP-GlcNAc and UDP-GlcNPr. The ratio of conversion rates (UDP-GlcNPr/UDP-GlcNAc) indicates the selectivity of the enzyme against UDP-GlcNPr.

To confirm the molecular docking results, mutagenesis analysis was performed for three key amino acids: Leu653, Thr560 and Met501. The first two amino acids were predicted to be responsible for forming an H-bond with the sugar moiety of UDP-GlcNAc, Thr560 via the side chain hydroxyl group, and Leu653 via its backbone carbonyl oxygen. The hOGT structure showed that Leu653 lies in a flexible loop region, suggesting a bulkier amino acid residue might potentially influence its spatial position, and break the H-bond between the carbonyl oxygen and C4-OH in the sugar donor. Based on this hypothesis, Leu653 was substituted by either amino acids with larger side chains (Tyr or Phe), or amino acids with small side chains (Val or Ile), while Thr560 was substituted by either a similar Ser or hydrophobic amino acid; e.g. Ala or Val. All the mutants (T560S, T560A, T560V, L653V, L653I, L653Y and L653F) showed decreased catalytic capability against UDP-GlcNAc in contrast to the wild type sOGT (Figure 4A). However, T560S showed higher catalytic capability compared with T560A or T560V, while L653V and L653I both showed higher enzymatic activity compared with L653Y or L653F. As Ser shares a similar side chain as that of Thr, T560S might still form an H-bond with UDP-GlcNAc via the hydroxyl group at C2, but T560A and T560V could not. For L653 mutants, an amino acid with a bulkier side chain (L653Y and L653F) seemed to be more influential on the spatial position of the backbone carbonyl oxygen. Combining these results with that of screening and Km values of non-hydroxyl analogs, it can be inferred that the H-bonds between these two amino acids and sugar donors might play significant roles in sOGT-sugar donor recognition.

Met501 has been predicted to be responsible for the steric hindrance. To decrease the steric hindrance between Met501 and the C2 N-acyl group, Met was substituted with amino acids with smaller side chains; i.e., Ala or Val. Both the mutants (M501A/M501V) showed decreased activity in contrast to the wild type sOGT, using either UDP-GlcNAc or UDP-GlcNPr as the sugar donors. However, the ratio of conversion rates (UDP-GlcNPr/UDP-GlcNAc), which indicates substrate selectivity of an enzyme, illustrated that both mutants were prone to use UDP-GlcNPr in contrast to UDP-GlcNAc, especially M501V (Figure 4B). Unfortunately, neither of the mutants showed broader substrate specificity when UDP-GlcNAc analogs substituted at C2 were used as sugar donors. The results indicate steric hindrance between M501 and the C2 N-acyl group is an influential factor in sOGT-sugar donor recognition. In a previous study of OGT inhibitors, it was also found that compounds with a short linker between the C2 N-acyl group and a fluorophore disturbed its binding to OGT, while a longer linker made it work, which is consistent with our results showing that the limit space impedes the binding of some analogs [37].

### Conclusions

O-GlcNAcylation is an important post-translational modification of proteins. OGT and OGA are the only two enzymes responsible for the sugar addition and removal, respectively. In this study, we investigated the sugar binding mechanism of sOGT and unraveled several influential factors in OGT-sugar donor recognition. This implicated that the backbone carbonyl oxygen of Leu653 and the hydroxyl group of Thr560, and especially the latter one, probably contributed to its binding to UDP-GlcNAc via hydrogen bonds. The close vicinity between Met501 and the N-acyl group and the hydrophobic environment around the N-acyl group were influential factors for sOGT recognition of some UDP-GlcNAc analogs. Our results are in keeping with recent studies [26], [27]. These results may help with the rational design of donor analogs or inhibitors, which could be used to detect *O*-GlcNAcylated proteins and elucidate their importance in cellular events.

## Materials and Methods

### Materials

Unless stated otherwise, all materials were purchased from Beijing Dingguo Changsheng Biotechnology Co. Ltd., China. CKII peptide (KKKYPGGSTPVSSANMM; purity>95%) and octapeptide (YAVVPVSK; purity>95%) were synthesized by GL Biochem (Shanghai) Ltd., China [17], [21]. UDP-GlcNAc and its analogs, including UDP-6-deoxy-GlcNAc, UDP-GalNAc, UDP-4-deoxy-GlcNAc, UDP-6-deoxy-GalNAc, UDP-GlcNBu and UDP-GlcNPr, were prepared as previously described [38]. The synthesis of other UDP-GlcNAc analogs has been reported previously [39], [40].

### Cloning, Expression and Purification of sOGT

The human mOGT gene (GI: 2266993) codon optimized for *E. coli* heterologous expression was synthesized by GeneArt (Germany) and was cloned into pMA vector. The gene encoding sOGT was amplified from the constructed vector using the forward primer 5′-CCGGAATTCATGCATTATAAAGAAGCC-3′ and reverse primer 5′-ACGCGTCGACTGCGCTTTCGGTAACTT-3′ with restriction sites underlined. The sOGT gene was subsequently inserted into pET-28a between *EcoR* I and *Sal* I (Thermo Scientific, Life Science Research, FastDigest, USA). The recombinant vector was transformed into *E. coli* BL21 (DE3) to obtain a fusion protein with a C-terminal and N-terminal His-tag. For protein expression, 20 mL of LB medium (10 g tryptone, 5 g yeast extract and 10 g NaCl per liter) containing 35 µg/mL kanamycin was inoculated with a colony picked from the plate and grown at 37°C and 250 r.p.m. overnight. The culture was used to inoculate 1 L of LB medium that was induced with isopropyl β-D-1-thiogalactopyranoside at a final concentration of 0.05 mM until OD_600nm_ reached 0.4–0.6, and grown at 13°C and 110 r.p.m. for 20 h. Cells were harvested by centrifugation at 8,000×g for 10 min and suspended in lysis buffer (20 mM Tris-HCl, pH 7.4, 0.3 M NaCl, and 0.1% Triton-X 100). The cells were disrupted by ultra-sonication using a microtip with 45% power for 20 min (2 sec on and 4 sec off) on ice and applied to centrifugation (13000×g for 30 min) to remove precipitants. The supernatants of bacterial cell lysates were loaded onto a nickel affinity chromatography column packed with 6 mL Ni-NTA agarose (QIAGEN GmbH, Hilden, Germany), which was balanced with equilibrium buffer (20 mM Tris-HCl, pH 7.4, 0.3 M NaCl and 0.1% dithiothreitol). After washing unbounded proteins with washing buffer (30 mM, 50 mM, 80 mM and 100 mM imidazole, respectively, in 20 mM Tris-HCl, pH 7.4, 0.3 M NaCl, and 0.1% dithiothreitol), the fusion proteins were eluted with elution buffer (250 mM imidazole in 20 mM Tris-HCl, pH 7.4, 0.3 M NaCl, and 0.1% dithiothreitol). The purified protein was concentrated using a 30 kD Amicon® Ultra Centrifugal Filter Unit (Millipore, Ireland), and the buffer was changed to reaction buffer (125 mM NaCl, 1 mM EDTA, 2.5 mM THP, 20 mM Tris-HCl, pH 7.4) to remove imidazole. Protein expression and purification were analyzed by 12% SDS-PAGE. The protein concentration was determined by the Bradford method.

### Screening Assay

The reactions were performed at 37°C for 45 min, in a total volume of 100 µL containing 200 µM CKII peptide, 1 mM UDP-GlcNAc analogs, 76 µg sOGT, 12.5 mM MgCl_2_ and buffer (125 mM NaCl, 1 mM EDTA, 2.5 mM THP, 20 mM Tris-HCl, pH 7.4). After quenching by adding an equal volume of methanol, the reaction mixtures were centrifuged at 12, 000×g for 30 min and filtered with a 0.22 µm filter. The reaction mixture (40 µL) was loaded onto a C-18 reverse-phase chromatographic column to quantify the product. The yield was analyzed based on the integrated areas of products and acceptor substrate. Each reaction was repeated at least three times.

### Enzyme Assays

The reactions for kinetic measurements of the active sugar donor substrates were performed as described above, except that the mixture was incubated at 37°C for 30 min. The apparent kinetic parameters of active sugar donor substrates were obtained by varying UDP-GlcNAc analogs from 2.0 µM to 200.0 µM (2.0 µM, 5.0 µM, 10.0 µM, 20.0 µM, 30.0 µM, 50.0 µM, 100.0 µM and 200.0 µM) at a fixed concentration of CKII (600 µM). All reactions were then quenched by the addition of an equal volume of methanol and analyzed by HPLC, using UDP-GlcNAc as a positive control and the mixture without enzymes as a negative control. The apparent kinetic parameters were obtained by fitting the data into the Michaelis-Menten equation using Graphpad Prism 5 (LA Jolla, CA, USA). Data were expressed as mean±SD of triplicate samples from independent experiments.

### Molecular Docking

AutoDock 4.2 was used to perform molecular docking [41]. We re-docked the co-crystal ligands of human O-GlcNAc transferase complex (PDB ID: 3PE4) as a training set to get rational docking parameters [21]. The ligand UDP-GlcNAc was sketched in Sybyl-X 2.0 (Tripos, Certara Inc., USA), and 30 rounds of simulated annealing (200–700 k) was performed to find reasonable conformation for docking. Docking experiments were performed with the following parameters: grid spacing was 0.375 Å, the number of points in each dimension was set to 50, 48 and 60, the grid center was set to −19.364, 28.687 and 8.804 to make sure that whole binding pocket could be covered. Docking simulations were done using the Lamarckian genetic algorithm with: GA runs = 250, population size = 200, quaternion = 30.0 and torsion = 30.0. Other parameters were set to the default. The results were evaluated following User Guide for AutoDock 4.2 [42].

### Site-directed Mutagenesis

Mutation sites were predicted by molecular docking. All position information in the present work refers to the hOGT isoform 1 (UniProt O15294-3). Mutations were performed using an Easy Mutagenesis System (Beijing TransGen Biotech Co. China) according to the manufacturer’s instructions. All mutation primers are shown in Table 3. All mutants were proofed by DNA sequencing.

**Table 3:** Site mutation primers used in this study.

Mutation Site*	Forward Primer**	Reverse Primer**
**M501A**	5′CATAGCGCGCTGTATCCGCTGTCTC3′	5′ATACAGCGCGCTATGATGCGGATGA3′
**M501V**	5′CATAGCGTGCTGTATCCGCTGTCTC3′	5′ATACAGCACGCTATGATGCGGATGA3′
**T560A**	5′CATCCGGCCAGCCATCTGATGCAGA3′	5′ATGGCTGGCCGGATGATTACCAAAA3′
**T560V**	5′CATCCGGTCAGCCATCTGATGCAGA3′	5′ATGGCTGACCGGATGATTACCAAAA3′
**T560S**	5′CATCCGAGCAGCCATCTGATGCAGA3′	5′ATGGCTGCTCGGATGATTACCAAAA3′
**L653F**	5′ATGTGGTTCGGTTATCCGGGTACAA3′	5′ATAACCGAACCACATTGCCTGAATC3′
**L653Y**	5′ATGTGGTACGGTTATCCGGGTACAA3′	5′ATAACCGTACCACATTGCCTGAATC3′
**L653V**	5′ATGTGGGTGGGTTATCCGGGTACAA3′	5′ATAACCCACCCACATTGCCTGAATC3′
**L653I**	5′ATGTGGATAGGTTATCCGGGTACAA3′	5′ATAACCTATCCACATTGCCTGAATC3′

* Corresponding to hOGT isoform 1.

** Underlined base(s) indicates the mutation sites.

## References

[1] Torres CR, Hart GW (1984) Topography and polypeptide distribution of terminal N-acetylglucosamine residues on the surfaces of intact lymphocytes. Evidence for O-linked GlcNAc. J Biol Chem 259: 3308–3317. 6421821

[2] Hart GW, Housley MP, Slawson C (2007) Cycling of O-linked beta-N-acetylglucosamine on nucleocytoplasmic proteins. Nature 446: 1017–1022. 17460662 10.1038/nature05815

[3] Wells L, Vosseller K, Hart GW (2001) Glycosylation of nucleocytoplasmic proteins: signal transduction and O-GlcNAc. Science 291: 2376–2378. 11269319 10.1126/science.1058714

[4] Zachara NE, Hart GW (2006) Cell signaling, the essential role of O-GlcNAc! Biochim Biophys Acta. 1761: 599–617. 10.1016/j.bbalip.2006.04.007 16781888

[5] Butkinaree C, Park K, Hart GW (2010) O-linked beta-N-acetylglucosamine (O-GlcNAc): Extensive crosstalk with phosphorylation to regulate signaling and transcription in response to nutrients and stress. Biochimica Et Biophysica Acta-General Subjects 1800: 96–106. 10.1016/j.bbagen.2009.07.018 PMC2815129 19647786

[6] Comer FI, Hart GW (1999) O-GlcNAc and the control of gene expression. Biochim Biophys Acta 1473: 161–171. 10580136 10.1016/s0304-4165(99)00176-2

[7] Rexach JE, Clark PM, Mason DE, Neve RL, Peters EC, et al (2012) Dynamic O-GlcNAc modification regulates CREB-mediated gene expression and memory formation. Nat Chem Biol 8: 253–261. 22267118 10.1038/nchembio.770 PMC3288555

[8] Kanno T, Yaguchi T, Nagata T, Mukasa T, Nishizaki T (2010) Regulation of AMPA receptor trafficking by O-glycosylation. Neurochem Res 35: 782–788. 20165912 10.1007/s11064-010-0135-1

[9] Mi W, Gu Y, Han C, Liu H, Fan Q, et al (2011) O-GlcNAcylation is a novel regulator of lung and colon cancer malignancy. Biochim Biophys Acta 1812: 514–519. 21255644 10.1016/j.bbadis.2011.01.009

[10] Krzeslak A, Forma E, Bernaciak M, Romanowicz H, Brys M (2012) Gene expression of O-GlcNAc cycling enzymes in human breast cancers. Clin Exp Med 12: 61–65. 21567137 10.1007/s10238-011-0138-5 PMC3295997

[11] Lynch TP, Ferrer CM, Jackson SR, Shahriari KS, Vosseller K, et al (2012) Critical role of O-Linked beta-N-acetylglucosamine transferase in prostate cancer invasion, angiogenesis, and metastasis. J Biol Chem 287: 11070–11081. 22275356 10.1074/jbc.M111.302547 PMC3322861

[12] Akimoto Y, Hart GW, Wells L, Vosseller K, Yamamoto K, et al (2007) Elevation of the post-translational modification of proteins by O-linked N-acetylglucosamine leads to deterioration of the glucose-stimulated insulin secretion in the pancreas of diabetic Goto-Kakizaki rats. Glycobiology 17: 127–140. 17095531 10.1093/glycob/cwl067

[13] Griffith LS, Schmitz B (1995) O-linked N-acetylglucosamine is upregulated in Alzheimer brains. Biochem Biophys Res Commun 213: 424–431. 7646495 10.1006/bbrc.1995.2149

[14] Hart GW, Slawson C, Ramirez-Correa G, Lagerlof O (2011) Cross talk between O-GlcNAcylation and phosphorylation: roles in signaling, transcription, and chronic disease. Annu Rev Biochem 80: 825–858. 21391816 10.1146/annurev-biochem-060608-102511 PMC3294376

[15] Hanover JA, Yu S, Lubas WB, Shin SH, Ragano-Caracciola M, et al (2003) Mitochondrial and nucleocytoplasmic isoforms of O-linked GlcNAc transferase encoded by a single mammalian gene. Arch Biochem Biophys 409: 287–297. 12504895 10.1016/s0003-9861(02)00578-7

[16] Kreppel LK, Hart GW (1999) Regulation of a cytosolic and nuclear O-GlcNAc transferase. Role of the tetratricopeptide repeats. J Biol Chem 274: 32015–32022. 10542233 10.1074/jbc.274.45.32015

[17] Lubas WA, Hanover JA (2000) Functional expression of O-linked GlcNAc transferase - Domain structure and substrate specificity. Journal of Biological Chemistry 275: 10983–10988. 10753899 10.1074/jbc.275.15.10983

[18] Iyer SPN, Hart GW (2003) Roles of the tetratricopeptide repeat domain in O-GlcNAc transferase targeting and protein substrate specificity. Journal of Biological Chemistry 278: 24608–24616. 12724313 10.1074/jbc.M300036200

[19] Kreppel LK, Blomberg MA, Hart GW (1997) Dynamic glycosylation of nuclear and cytosolic proteins. Cloning and characterization of a unique O-GlcNAc transferase with multiple tetratricopeptide repeats. J Biol Chem 272: 9308–9315. 9083067 10.1074/jbc.272.14.9308

[20] Lazarus BD, Roos MD, Hanover JA (2005) Mutational analysis of the catalytic domain of O-linked N-acetylglucosaminyl transferase. Journal of Biological Chemistry 280: 35537–35544. 16105839 10.1074/jbc.M504948200

[21] Lazarus MB, Nam Y, Jiang J, Sliz P, Walker S (2011) Structure of human O-GlcNAc transferase and its complex with a peptide substrate. Nature 469: 564–567. 21240259 10.1038/nature09638 PMC3064491

[22] Wrabl JO, Grishin NV (2001) Homology between O-linked GlcNAc transferases and proteins of the glycogen phosphorylase superfamily. J Mol Biol 314: 365–374. 11846551 10.1006/jmbi.2001.5151

[23] Jinek M, Rehwinkel J, Lazarus BD, Izaurralde E, Hanover JA, et al (2004) The superhelical TPR-repeat domain of O-linked GlcNAc transferase exhibits structural similarities to importin alpha. Nat Struct Mol Biol 11: 1001–1007. 15361863 10.1038/nsmb833

[24] Martinez-Fleites C, Macauley MS, He Y, Shen DL, Vocadlo DJ, et al (2008) Structure of an O-GlcNAc transferase homolog provides insight into intracellular glycosylation. Nat Struct Mol Biol 15: 764–765. 18536723 10.1038/nsmb.1443

[25] Clarke AJ, Hurtado-Guerrero R, Pathak S, Schuttelkopf AW, Borodkin V, et al (2008) Structural insights into mechanism and specificity of O-GlcNAc transferase. Embo Journal 27: 2780–2788. 18818698 10.1038/emboj.2008.186 PMC2556091

[26] Lazarus MB, Jiang J, Gloster TM, Zandberg WF, Whitworth GE, et al (2012) Structural snapshots of the reaction coordinate for O-GlcNAc transferase. Nat Chem Biol 8: 966–968. 23103939 10.1038/nchembio.1109 PMC3508357

[27] Schimpl M, Zheng X, Borodkin VS, Blair DE, Ferenbach AT, et al (2012) O-GlcNAc transferase invokes nucleotide sugar pyrophosphate participation in catalysis. Nat Chem Biol 8: 969–974. 23103942 10.1038/nchembio.1108 PMC3509171

[28] Lee SS, Hong SY, Errey JC, Izumi A, Davies GJ, et al (2011) Mechanistic evidence for a front-side, SNi-type reaction in a retaining glycosyltransferase. Nat Chem Biol 7: 631–638. 21822275 10.1038/nchembio.628

[29] Mayer A, Gloster TM, Chou WK, Vocadlo DJ, Tanner ME (2011) 6 ′′-Azido-6 ′′-deoxy-UDP-N-acetylglucosamine as a glycosyltransferase substrate. Bioorg Med Chem Lett 21: 1199–1201. 21273069 10.1016/j.bmcl.2010.12.090

[30] Gurcel C, Vercoutter-Edouart AS, Fonbonne C, Mortuaire M, Salvador A, et al (2008) Identification of new O-GlcNAc modified proteins using a click-chemistry-based tagging. Anal Bioanal Chem 390: 2089–2097. 18369606 10.1007/s00216-008-1950-y

[31] Vocadlo DJ, Hang HC, Kim EJ, Hanover JA, Bertozzi CR (2003) A chemical approach for identifying O-GlcNAc-modified proteins in cells. Proc Natl Acad Sci U S A 100: 9116–9121. 12874386 10.1073/pnas.1632821100 PMC171382

[32] Boyce M, Carrico IS, Ganguli AS, Yu SH, Hangauer MJ, et al (2011) Metabolic cross-talk allows labeling of O-linked beta-N-acetylglucosamine-modified proteins via the N-acetylgalactosamine salvage pathway. Proc Natl Acad Sci U S A 108: 3141–3146. 21300897 10.1073/pnas.1010045108 PMC3044403

[33] Lubas WA, Smith M, Starr CM, Hanover JA (1995) Analysis of nuclear pore protein p62 glycosylation. Biochemistry 34: 1686–1694. 7849028 10.1021/bi00005a025

[34] Shen DL, Gloster TM, Yuzwa SA, Vocadlo DJ (2012) Insights into O-linked N-acetylglucosamine ([0–9]O-GlcNAc) processing and dynamics through kinetic analysis of O-GlcNAc transferase and O-GlcNAcase activity on protein substrates. J Biol Chem 287: 15395–15408. 22311971 10.1074/jbc.M111.310664 PMC3346082

[35] Vosseller K, Trinidad JC, Chalkley RJ, Specht CG, Thalhammer A, et al (2006) O-linked N-acetylglucosamine proteomics of postsynaptic density preparations using lectin weak affinity chromatography and mass spectrometry. Mol Cell Proteomics 5: 923–934. 16452088 10.1074/mcp.T500040-MCP200

[36] Wang Z, Udeshi ND, Slawson C, Compton PD, Sakabe K, et al (2010) Extensive crosstalk between O-GlcNAcylation and phosphorylation regulates cytokinesis. Sci Signal 3: ra2. 20068230 10.1126/scisignal.2000526 PMC2866299

[37] Gross BJ, Kraybill BC, Walker S (2005) Discovery of O-GlcNAc transferase inhibitors. J Am Chem Soc 127: 14588–14589. 16231908 10.1021/ja0555217

[38] Cai L, Guan W, Kitaoka M, Shen J, Xia C, et al.. (2009) A chemoenzymatic route to N-acetylglucosamine-1-phosphate analogues: substrate specificity investigations of N-acetylhexosamine 1-kinase. Chem Commun (Camb): 2944–2946. 10.1039/b904853g 19436918

[39] Chen Y, Thon V, Li Y, Yu H, Ding L, et al (2011) One-pot three-enzyme synthesis of UDP-GlcNAc derivatives. Chem Commun (Camb) 47: 10815–10817. 21863157 10.1039/c1cc14034e PMC11279325

[40] Muthana MM, Qu J, Li Y, Zhang L, Yu H, et al (2012) Efficient one-pot multienzyme synthesis of UDP-sugars using a promiscuous UDP-sugar pyrophosphorylase from Bifidobacterium longum (BLUSP). Chem Commun (Camb) 48: 2728–2730. 22306833 10.1039/c2cc17577k

[41] Oliveira FG, Sant’Anna CM, Caffarena ER, Dardenne LE, Barreiro EJ (2006) Molecular docking study and development of an empirical binding free energy model for phosphodiesterase 4 inhibitors. Bioorg Med Chem 14: 6001–6011. 16843671 10.1016/j.bmc.2006.05.017

[42] Morris G, Goodsell D, Pique M, Lindstrom W, Huey R, et al (2012) User Guide AutoDock Version 4.2. Available: http://autodock.scripps.edu/faqs-help/manual/autodock-4-2-user-guide/AutoDock4.2_UserGuide.pdf. Accessed 2013 Mar 29.

[43] DeLano WL, Sarina Bromberg (2004) PyMOL User’s Guide. Available: http://pymol.sourceforge.net/newman/userman.pdf. Accessed 2013 Feb 28.

